# Potentially preventable unplanned hospitalizations due to adverse events in patients receiving systemic anticancer therapy: a retrospective single-center study

**DOI:** 10.1007/s00520-026-11351-0

**Published:** 2026-10-13

**Authors:** Momoka Endo, Hirotoshi Iihara, Hironori Fujii, Daichi Watanabe, Shuji Sakurai, Atsuko Tomida, Masashi Kuno, Kyoko Nakano, Masahito Shimizu, Keisuke Kumada, Akio Suzuki

**Affiliations:** 1 Department of Pharmacy, Gifu University Hospital, 1-1 Yanagido, Gifu, Gifu 501-1194 Japan; 2 Patient Safety Division, Gifu University Hospital, 1-1 Yanagido, Gifu, Gifu 501-1194 Japan; 3 Practical Pharmaceutical Research Center, Gifu Pharmaceutical University, 1-25-4 Daigaku-Nishi, Gifu, Gifu 501-1196 Japan; 4 Department of Gastroenterological Surgery and Pediatric Surgery, Gifu University Graduate School of Medicine, 1-1 Yanagido, Gifu, Gifu 501-1194 Japan; 5 Department of Nursing, Gifu University Hospital, 1-1 Yanagido, Gifu, Gifu 501-1194 Japan; 6 Department of Gastroenterology, Gifu University Graduate School of Medicine, 1-1 Yanagido, Gifu, Gifu 501-1194 Japan; 7 Laboratory of Advanced Medical Pharmacy, Gifu Pharmaceutical University, 1-25-4 Daigakunishi, Gifu, Gifu, 501-1196 Japan

**Keywords:** Systemic anticancer therapy, Unplanned hospitalization, Supportive care, Adverse events

## Abstract

**Purpose:**

Unplanned hospitalizations during systemic anticancer therapy remain a major clinical challenge. This study aimed to investigate the causes of unplanned hospitalizations associated with systemic anticancer therapy-related adverse events and to assess their potential preventability.

**Methods:**

We conducted a single-center retrospective analysis of unplanned hospitalizations due to adverse events related to systemic anticancer therapy between March 1, 2023, and March 31, 2024. The type, severity, and treatment of adverse events leading to unplanned hospitalization were reviewed. The preventability of these hospitalizations was assessed by a multidisciplinary team (pharmacists, physicians, and nurses) based on domestic and international guidelines and previous reports.

**Results:**

A total of 161 cases of unplanned hospitalizations due to systemic anticancer therapy-related adverse events were identified. Among these, 30 cases (18.6%) were considered potentially preventable. The types of potentially preventable interventions included optimization of supportive care, such as G-CSF prophylaxis, antimicrobial prophylaxis, and antiemetic optimization (15 cases); optimization of anticancer therapy, including dose reduction/modification (6 cases); early detection and management, including enhanced monitoring and patient education (6 cases); and preventive measures before anticancer therapy, including varicella-zoster virus vaccination (5 cases).

**Conclusions:**

Approximately 20% of unplanned hospitalizations related to systemic anticancer therapy were potentially preventable. Optimization of supportive care and enhancement of medical care systems could reduce unplanned hospitalizations.

## Introduction

The recent use of molecularly targeted agents and immune checkpoint inhibitors, together with advances in supportive care, has significantly improved outcomes in systemic anticancer therapy and led to enhanced survival and treatment response [1–3]. Nevertheless, treatment-related adverse events remain common and continue to pose a major clinical challenge. Serious adverse events are associated with impaired quality of life (QOL), treatment interruption, and increased risk of short-term mortality [4, 5]. Furthermore, as systemic anticancer therapy is increasingly administered in the outpatient setting, appropriate management of adverse events, including patient education, has become essential.

Unplanned hospitalization is recognized as an important indicator of the quality of adverse event management during systemic anticancer therapy [6]. Cohort studies have reported that approximately 25% of patients initiating systemic anticancer therapy require acute care, including emergency department visits or unplanned hospitalizations, within 30 days of treatment initiation [7, 8]. The major causes of unplanned hospitalizations include febrile neutropenia (FN), infections, and gastrointestinal toxicities [9, 10]. These events impose substantial physical and psychological burdens on patients and increase healthcare resource utilization [11]. Therefore, reducing preventable unplanned hospitalizations is an important strategy for improving the quality and efficiency of oncology care [6].

In recent years, the importance of supportive care in systemic anticancer therapy has been emphasized, and evidence-based guidelines have been established for the use of prophylactic granulocyte colony-stimulating factor (G-CSF) for FN prevention [12], optimization of antiemetic therapy [13, 14], and infection prevention strategies [15]. However, guideline-recommended supportive measures are not consistently implemented [16].

Internationally, attention has increasingly focused on evaluating the preventability of unplanned hospitalizations associated with systemic anticancer therapy [10]. Large administrative database studies in the United States have estimated that approximately 5–7% of systemic anticancer therapy-related hospitalizations may be potentially preventable [10]. Moreover, detailed clinical chart review studies have suggested that avoidable systemic anticancer therapy-related hospitalizations account for 21% of cases among patients with gastrointestinal malignancies, and may be preventable through selective chemotherapy use and aggressive management of adverse effects [17]. However, most evidence to date has been derived from Western countries and their applicability to Asian populations, including Japan, where healthcare delivery systems may differ, remains unclear. Accordingly, real-world data for these populations based on detailed clinical assessment are limited, and the characteristics and preventability of systemic anticancer therapy-related unplanned hospitalizations are not fully understood.

Here, to identify potentially preventable unplanned hospitalizations associated with adverse events related to systemic anticancer therapy, we retrospectively analyzed unplanned hospitalizations occurring within 30 days after treatment at our institution. We examined the type, severity, and management of adverse events leading to hospitalization and assessed their preventability based on guideline-recommended interventions through multidisciplinary chart review.

## Methods

### Study design and case selection

This study was conducted as a single-center, retrospective observational study. All unplanned hospitalizations at our hospital between March 1, 2023, and March 31, 2024, were included. Previous studies have evaluated outcomes within 30 days after the last administration of systemic anticancer therapy [18, 19]. Accordingly, we defined the period within 30 days after the last administration of systemic anticancer therapy as “the post-systemic anticancer therapy period”.

Unplanned hospitalization was defined, in accordance with previous reports, as an unscheduled hospital admission [20]. Planned admissions, including scheduled chemotherapy, elective surgery, and planned transfers to our institution, were excluded. Hospitalizations due to disease progression and emergency transfers were included in the screening process, whereas cases managed solely in the emergency department without hospital admission were excluded.

Cases were extracted by retrieving the list of unplanned hospital admissions from the electronic medical record system (HOPE EGMAIN-GX; Fujitsu Ltd., Tokyo, Japan) and reviewing the administration history and reason for admission.

All unplanned hospitalizations occurring during the study period were screened, and hospitalizations involving patients who had received systemic anticancer drug therapy were identified. Medical records were reviewed to determine whether each unplanned hospitalization was associated with an adverse event related to systemic anticancer drug therapy. Multiple unplanned hospitalizations in the same patient were treated as independent events in the analysis.

### Data collection

The following items were retrospectively extracted from the electronic medical records for each unplanned hospitalization.

- 1) Patient characteristics: Age, sex, primary tumor site, performance status (PS), and comorbidities.
- 2) Laboratory data at the time of unplanned hospitalization: Total protein, serum albumin, aspartate aminotransferase (AST), alanine aminotransferase (ALT), total bilirubin, serum creatinine, C-reactive protein (CRP), white blood cell count, neutrophil count, platelet count, and hemoglobin.
- 3) Treatment-related factors: Systemic anticancer therapy regimen, number of treatment cycles, prior treatment history, and dose modifications of the regimen.
- 4) Adverse event-related items: Type of adverse event leading to unplanned hospitalization, severity (assessed based on the Common Terminology Criteria for Adverse Events (CTCAE), version 5.0), onset timing (number of days from last treatment day), hospital management (supportive care, antimicrobial administration, blood transfusion, etc.), and length of hospitalization.

### Assessment of preventability of unplanned hospitalizations

The preventability of unplanned hospitalizations was evaluated using the following process. Preventability was assessed by determining whether the unplanned hospitalization could reasonably have been avoided through guideline-concordant interventions implemented before the onset of the adverse event, based on clinical information available at that time.

Evaluation criteria were established based on domestic and international guidelines and previous reports, focusing on opportunities for preventive intervention from the following perspectives:

- ‧ Indications for primary and secondary prophylactic G-CSF administration [12]
- ‧ Optimization of supportive therapies such as antiemetic therapy [13]
- ‧ Indications for prophylactic antimicrobial administration [15]
- ‧ Dose adjustment based on package inserts and treatment protocols for each regimen
- ‧ Preventive measures such as vaccination [21]
- ‧ Strengthening of early monitoring and outpatient follow-up systems [22, 23]

Following this assessment, a case was classified as “potentially preventable” if at least one of the following conditions was met:

- 1. Omission of a guideline-recommended prophylactic intervention
- 2. Failure to modify treatment intensity despite known patient-specific risk factors
- 3. Delayed assessment or intervention after early symptom presentation

To assess the causal relationship between unplanned hospitalizations and systemic anticancer therapy, two pharmacists independently reviewed each case based on the medical records at admission, the timing of adverse event onset, and clinical course. Because this was a retrospective medical record review, the assessors were not blinded to the clinical outcomes and reviewed the medical records in their entirety. In cases of disagreement, consensus was reached through discussion between the two pharmacists.

Cases determined to be associated with systemic anticancer therapy were subsequently reviewed by a multidisciplinary team consisting of two pharmacists, one physician, and one nurse to assess the potential for preventive intervention based on the predefined criteria. Evaluators reviewed each case’s medical records, treatment regimen, and clinical course of the adverse event. Preventability was judged using the clinical information available before the onset of the adverse event.

Each hospitalization was classified as either potentially preventable or non-preventable. Cases with discordant assessments were reviewed in structured multidisciplinary case conferences according to the predefined assessment framework, and the final classification was determined by consensus. For cases classified as potentially preventable, the specific preventive interventions were documented.

A study-specific assessment framework based on relevant clinical guidelines and previous reports was used rather than a previously validated instrument specifically developed for this clinical context.

### Classification of potentially preventable cases

Potentially preventable cases were classified into the following categories based on the type of preventive intervention:

- ‧ Optimization of supportive care (e.g., G-CSF prophylaxis, antimicrobial prophylaxis, antiemetic optimization, and optimization of comorbidity management)
- ‧ Optimization of anticancer therapy, including dose reduction/modification
- ‧ Early detection and management, including enhanced monitoring and patient education
- ‧ Preventive measures before anticancer therapy, such as vaccination (e.g., herpes zoster vaccination)

Vaccination was classified separately because it represents a preventive intervention implemented before the initiation of systemic anticancer therapy. Cases falling into multiple categories were counted in each respective category.

The primary outcome of this study was the proportion of unplanned hospitalizations classified as potentially preventable.

### Statistical analysis

Patient characteristics and laboratory data were summarized using medians (interquartile ranges) for continuous variables and frequencies (percentages) for categorical variables. The proportion of potentially preventable hospitalizations was reported with a 95% confidence interval calculated using the Wilson score method. Each adverse event category was compared between potentially preventable and non-preventable hospitalizations using a two-sided Fisher's exact test. P values were not adjusted for multiple comparisons. *P* < 0.05 was considered statistically significant. Statistical analyses were performed using R version 4.5.3.

### Ethics statement

This study was approved by the Ethics Committee of Gifu University School of Medicine in 2025 (approval no. 2025–040). All procedures involving human participants were conducted in accordance with the ethical standards of the institutional and/or national research committee and with the 1964 Helsinki Declaration and its later amendments or comparable ethical standards. This study was a retrospective study that utilized existing medical records only. All data were handled anonymously, with care taken to prevent individual identification.

### Consent to participate

Because this was a retrospective observational study using only existing medical records, the requirement for written informed consent was waived by the Ethics Committee of Gifu University School of Medicine. Information regarding participation in the study was posted on our hospital website using an opt-out method, ensuring that research subjects had the opportunity to decline participation.

## Results

### Patient characteristics

**Fig. 1:**
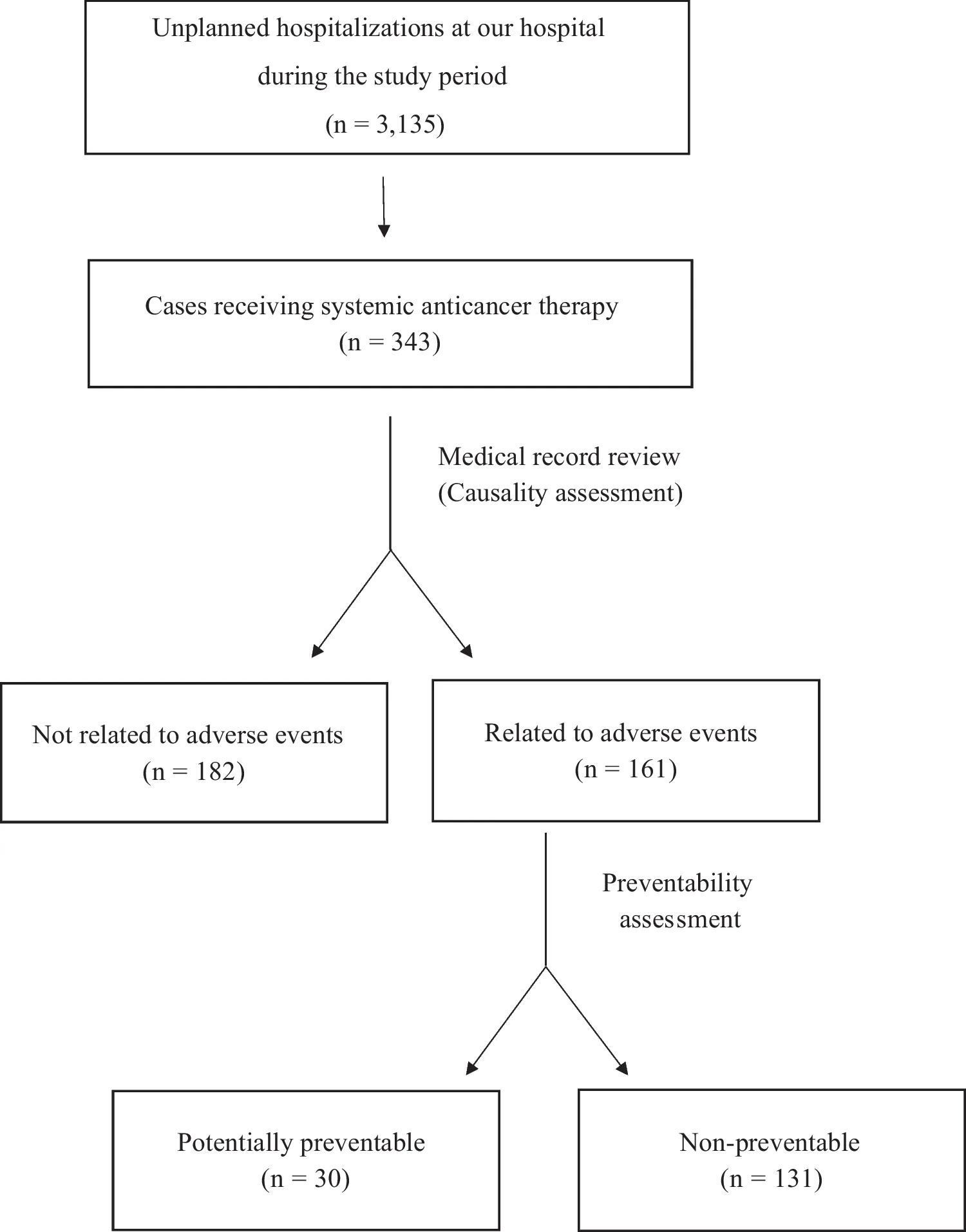
Flowchart for identification of cases for analysis

A total of 3,135 unplanned hospitalizations occurred at our hospital between March 1, 2023, and March 31, 2024. Among them, 343 cases (10.9%) were undergoing systemic anticancer therapy, and 161 (46.9%) were associated with adverse events related to systemic anticancer therapy, accounting for 5.1% of all unplanned hospitalizations (Fig. 1).

Table 1 shows the characteristics of the 161 cases at the time of unplanned hospitalization. These included 67 involving male patients (41.6%) and 94 involving female patients (58.4%), with a median age of 68 years (range: 5–92 years). The median values for laboratory parameters were serum albumin 3.2 g/dL (interquartile range: 2.9–3.9), CRP 4.79 mg/dL (interquartile range: 1.03–13.54), and neutrophil count 3,210/μL (interquartile range: 1,505–6,527).

**Table 1:** Characteristics of cases with unplanned hospitalization (*n* = 161)

Sex, male/female, n	67/94
Age, median (range), years	68 (5–92)
Laboratory data, median (IQR)	
Total protein (g/dL)	6.2 (4.2–8.3)
Serum albumin (g/dL)	3.2 (2.9–3.9)
Aspartate aminotransferase (IU/L)	28 (19–42)
Alanine aminotransferase (IU/L)	21 (15–35)
Total bilirubin (mg/dL)	0.7 (0.5–1.1)
Serum creatinine (mg/dL)	0.86 (0.67–1.22)
C-reactive protein (mg/dL)	4.79 (1.03–13.54)
White blood cell count (/μL)	5,050 (2,360–8,700)
Neutrophil count (/μL)	3,210 (1,505–6,527)
Platelet count (× 10^9^/L)	166 (105–238)
Hemoglobin (g/dL)	10.6 (9.1–12.2)

*IQR* Interquartile Range

### Classification of adverse events leading to unplanned hospitalization

Table 2 shows the adverse events leading to unplanned hospitalization among the 161 cases.

**Table 2:** Classification of adverse events leading to unplanned hospitalization (*n* = 161)

Type of adverse event	Overall (*n* = 161)	Potentially preventable (*n* = 30)	Non-preventable (*n* = 131)	*P* Value
Infections associated with treatment-related immunosuppression (excluding FN and pneumonia)^a^	40 (24.8)	5 (16.7)	35 (26.7)	0.349
FN	23 (14.3)	10 (33.3)	13 (9.9)	0.003
Fever/fatigue^b^	22 (13.7)	0 (0.0)	22 (16.8)	0.015
Pneumonia	20 (12.4)	4 (13.3)	16 (12.2)	0.769
Gastrointestinal symptoms^c^	14 (8.7)	6 (20.0)	8 (6.1)	0.026
irAE	9 (5.6)	0 (0.0)	9 (6.9)	0.211
Others	33 (20.5)	5 (16.7)	28 (21.4)	0.802

*FN* Febrile neutropenia, *irAE* Immune-related adverse events

^a^e.g., enteritis, cholangitis, herpes zoster, COVID-19

^b^Cases with an infectious source identified after further evaluation were excluded from the fever category

^c^e.g., nausea, vomiting, and diarrhea

The most frequent events were infections (excluding FN and pneumonia) that occurred during systemic anticancer therapy and were suggestive of treatment-related immunosuppression (e.g., enteritis, cholangitis, herpes zoster, COVID-19), accounting for 40 cases (24.8%). This was followed by FN in 23 cases (14.3%), fever/fatigue in 22 cases (13.7%), pneumonia in 20 cases (12.4%), gastrointestinal symptoms (nausea, vomiting, and diarrhea) in 14 cases (8.7%), and immune-related adverse events (irAE) in 9 cases (5.6%).

### Assessment of preventability through preventive interventions

Following assessment by a multidisciplinary team, 30 (18.6%, 95% CI, 13.4–25.4%) of the 161 cases were determined to be potentially preventable through preventive interventions. Cases deemed potentially preventable were categorized by intervention type. Representative issues and specific examples are shown in Table 3. The most frequent category was optimization of supportive care, comprising 15 cases (50.0%), including 7 cases requiring optimization of G-CSF prophylaxis, 3 requiring optimization of prophylactic antimicrobial use, 4 requiring optimization of antiemetic therapy, and 1 requiring optimization of comorbidity management. This was followed by optimization of anticancer therapy in 6 cases (20.0%), early detection and management in 6 cases (20.0%), and preventive measures before anticancer therapy in 5 cases (16.7%). Multiple intervention types could be applied to a single case; therefore, the total number exceeded 30 cases.

**Table 3:** Classification of potentially preventable cases by intervention type (*n* = 30)

Intervention type	Cases (%) ^a^	Key issues	Representative examples
Optimization of supportive care	15 (50.0)		
・G-CSF prophylaxis	7	Omission of primary and secondary prophylaxis	No primary G-CSF prophylaxis for high-risk regimens (TC, M-VAC) (4 cases)
			No secondary prophylaxis after prior FN (1 case)
			Discharge despite severe neutropenia requiring consideration of G-CSF support (1 case)
			G-CSF prophylaxis was withheld because of patient refusal due to severe arthralgia after prior administration (1 case)
・Antimicrobial prophylaxis	3	Insufficient consideration of prophylaxis in high-risk patients	Full-dose VDC/IE was continued to maintain dose intensity despite prior FN; prophylactic antimicrobial therapy could have been considered because of persistent high risk of infection (1 case)
			No prophylactic antibiotics in a patient with multiple immunosuppressive factors (1 case)
・Antiemetic optimization	4	Inadequate antiemetic therapy according to CINV risk	Young women receiving moderately to highly emetogenic regimens (CCRT, TC, CapeOX) despite standard prophylaxis; additional olanzapine could have been considered (4 cases)
・Optimization of comorbidity management	1	Insufficient management of comorbidities before anticancer therapy	FOLFIRINOX was initiated despite poorly controlled diabetes (HbA1c 8.3%), resulting in hyperglycemia (1 case)
Optimization of anticancer therapy ・Dose reduction/modification	6 (20.0)	Insufficient consideration of renal function/PS	Standard-dose chemotherapy despite poor PS and renal dysfunction in an elderly patient (1 case)
		Failure to adhere to dose reduction criteria	Standard-dose chemotherapy continued in a patient with prior Grade 4 neutropenia (1 case)
			Treatment intensity not adjusted in a patient with poor PS (1 case)
			Failure to adhere to dose reduction criteria for peripheral neuropathy (1 case)
			Systemic anticancer therapy initiated despite poor baseline PS (1 case) ^b^
Early detection and management ・Enhanced monitoring and patient education	6 (20.0)	Insufficient additional assessment and outpatient follow-up	No SpO₂ measurement or imaging despite fever/elevated CRP (2 cases)
			Insufficient outpatient follow-up after early discharge following CAPOXIRI initiation (2 cases)
			Delay in endocrine consultation for endocrine irAEs (1 case)
Preventive measures before anticancer therapy ・Vaccination (e.g., herpes zoster vaccination)	5 (16.7)	Insufficient assessment of the need for herpes zoster vaccination	Herpes zoster during highly immunosuppressive regimens without prior vaccination assessment (5 cases)

*G-CSF* Granulocyte colony-stimulating factor, *TC* Docetaxel + cyclophosphamide, *M-VAC* Methotrexate + vinblastine + doxorubicin + cisplatin, *FN* Febrile neutropenia, *VDC* Vincristine + doxorubicin + cyclophosphamide, *IE* Ifosfamide + etoposide, *CINV* Chemotherapy-induced nausea and vomiting, *CCRT* Concurrent chemoradiotherapy, *CapeOX* Capecitabine + oxaliplatin, *PS* Performance status, *FOLFIRINOX* Fluorouracil + irinotecan + oxaliplatin + leucovorin, *CRP* C-reactive protein, *CAPOXIRI* Capecitabine + oxaliplatin + irinotecan, *irAE* Immune-related adverse events, *Nab-PTX* Albumin-bound paclitaxel, *R-CHOP* Rituximab + cyclophosphamide + doxorubicin + vincristine + prednisolone, *TMZ* temozolomide

^a^Cases could be assigned to multiple intervention categories; therefore, the total number of category assignments exceeds 30 (total = 32)

^b^ This patient had poor baseline PS and experienced early clinical worsening after systemic anticancer therapy initiation. The case was considered to be associated with systemic anticancer therapy and was included in the analysis

Among the 30 potentially preventable hospitalizations, febrile neutropenia was the most frequent adverse event (10 cases), followed by gastrointestinal symptoms (6 cases).

The remaining 131 cases (81.4%) were judged difficult to prevent through intervention due to factors such as deterioration of general condition accompanying disease progression, unpredictable serious adverse events, or adverse events occurring despite appropriate supportive therapy.

## Discussion

In this study, we retrospectively examined the potential preventability of unplanned hospitalizations associated with adverse events in 161 cases involving patients undergoing systemic anticancer therapy. Overall, 30 cases (18.6%) were considered potentially preventable, with suboptimal supportive care, inadequate dose adjustment, insufficient monitoring, and lack of preventive measures identified as potential contributors. These findings suggest that improving supportive care and adverse event management may reduce unplanned hospitalizations. Given the retrospective design of this study and its reliance on expert judgment, these results should be interpreted as hypothesis-generating.

Suboptimal supportive care accounted for the largest proportion of potentially preventable hospitalizations, consistent with Brooks et al. [17]. In particular, insufficient use of G-CSF for primary and secondary prophylaxis may have contributed to FN-related hospitalizations, whereas guideline-based risk-adapted use is known to reduce this risk [12, 24].

In addition, although standard antiemetic prophylaxis was administered according to the Japan Society of Clinical Oncology (JSCO) guidelines in this study, escalation based on nausea and vomiting in prior cycles was not implemented in some patients [25]. Moreover, risk stratification tools, such as those from the MyRisk trial, may support individualized antiemetic strategies in the first cycle [26], and some patients in this study may have required intensified prophylaxis based on their characteristics and prior history. Individualized escalation based on prior-cycle experience and patient-specific risk factors may therefore help reduce unplanned hospitalizations.

On the other hand, current guidelines do not recommend universal antimicrobial prophylaxis [15]. Nevertheless, risk-adapted prophylaxis may be considered in selected high-risk patients such as elderly patients, those with a history of severe neutropenia, or those with multiple overlapping immunosuppressive factors [27]. In our study, some unplanned hospitalizations in these high-risk patients might have been potentially preventable with appropriate antimicrobial prophylaxis.

Following suboptimal supportive care, optimization of anticancer therapy was the second most frequent category among potentially preventable hospitalizations. This finding indicates the importance of appropriate dose modifications based on patient characteristics, including renal function, age, and performance status [28, 29]. Furthermore, inadequate monitoring systems were also identified as contributing factors. In the broader adult inpatient population, recent meta-analyses have suggested that outpatient follow-up after discharge may be associated with reduced 30-day readmission rates [22, 23]. Our findings support the importance of timely assessment during symptom exacerbation and structured follow-up. Early follow-up strategies for high-risk patients, such as scheduled outpatient visits, telephone-based monitoring by healthcare professionals, and structured patient education, may facilitate early detection of symptom worsening and enable prompt intervention [30, 31].

Some cases in this study suggest that unplanned hospitalizations might have been potentially preventable through vaccination. For example, herpes zoster vaccination has demonstrated efficacy in immunocompromised cancer patients [32], indicating a potential role for preventive strategies in selected populations.

The present findings indicate that unplanned hospitalizations are influenced not only by treatment-related factors but also by gaps in healthcare system processes. Standardization of guideline-based supportive care remains a key challenge [31]. Outcomes might be further improved by strategies such as regimen-specific supportive care protocols, risk stratification, and multidisciplinary collaboration. This approach might also include a team-based care model incorporating electronic medical record alerts, pharmacist-led review, and nurse-led monitoring.

This study has several limitations. First, it was conducted under a single-center, retrospective design with a relatively small sample size and may have been influenced by institutional and regional factors. Second, irAEs occurring more than 30 days after immune checkpoint inhibitor administration were outside the predefined observation period and were therefore not included in the present analysis. Third, although preventability was assessed by a multidisciplinary team using predefined criteria and discordant assessments were resolved by consensus, the classification relied on retrospective clinical judgment, and the possibility of selection and information bias cannot be excluded. Individual pre-consensus assessments were not retained in a format suitable for inter-rater reliability analysis; therefore, inter-rater reliability could not be quantified. In addition, a study-specific assessment framework was used rather than a previously validated instrument specifically developed for this clinical context. These factors may limit the reproducibility and comparability of the preventability classification. Fourth, because the preventive interventions were not prospectively evaluated, preventability should be interpreted as an estimate rather than a definitive outcome.

## Conclusion

Approximately 20% of unplanned hospitalizations were considered potentially preventable. These hospitalizations may be reduced by optimization of supportive care, including G-CSF prophylaxis, prophylactic antimicrobial use, and antiemetic therapy, as well as appropriate dose adjustment, early monitoring, and preventive strategies such as vaccination. Future prospective studies are warranted to evaluate the effectiveness of these preventive strategies.

## Data Availability

The data that support the findings of this study are available from the corresponding author upon reasonable request.
